# An Assessment of the Knowledge and Awareness of Common Otorhinolaryngology-Related Issues Among School and University Students in Makkah City, Saudi Arabia: A Cross-Sectional Study

**DOI:** 10.7759/cureus.37444

**Published:** 2023-04-11

**Authors:** Mohammad S Jalaladdin, Eyad E Sindi, Abdulrahman F Kabli, Salah Bakry, Ammar K Mandili, Saad N Albagami, Mohammed I Alshinkity, Naif Abeeri, Osama Marglani

**Affiliations:** 1 Department of Medicine and Surgery, College of Medicine, Umm Al-Qura University, Makkah, SAU; 2 Department of Otolaryngology-Head and Neck Surgery, King Faisal Specialist Hospital and Research Center, Jeddah, SAU; 3 Department of Otolaryngology-Head and Neck Surgery, Faculty of Medicine, Umm Al-Qura University, Makkah, SAU

**Keywords:** saudi arabia, students, diseases, knowledge, attitude, otorhinolaryngology

## Abstract

Background

Otorhinolaryngology (ENT) illnesses commonly affect all individuals with a broad range of symptoms, and most causes are preventable. According to the WHO, more than 278 million people have bilateral hearing loss. Locally, a previously published study done in Riyadh showed that most participants (79.4%) had a poor level of knowledge on common ENT-related diseases. The current study aims to investigate and explore the knowledge of and attitudes toward common ENT problems among students in Makkah City, Saudi Arabia.

Method

This descriptive, cross-sectional study used an Arabic-language electronic questionnaire to assess knowledge of common ENT problems. It was distributed to medical students at Umm Al-Qura University and students attending Makkah City high schools in Saudi Arabia between November 2021 and October 2022. The sample size was calculated to be 385 participants.

Results

Overall 1080 respondents were enrolled in this survey from Makkah City. Participants with good knowledge of common ENT diseases were over 20 years of age with a p-value <0.001. Furthermore, females also had a significant p-value of <0.004, and those with bachelor's or university degrees showed a statistically significant p-value of <0.001.

Conclusion

Female participants with bachelor's or university degrees and participants aged 20 and older showed superior knowledge. Our findings indicate that students need educational implications and awareness campaigns to increase their knowledge, practice, and perception toward common otorhinolaryngology-related issues.

## Introduction

Otolaryngology (ENT) is the branch of medicine that deals with ear, nose, and throat disorders. ENT diseases are a significant public health problem that affects all age groups [1-3]. These may lead to impaired physiological functions in hearing, breathing, swallowing, phonation, speech, olfaction, taste, protection of the lower respiratory tract, and clearance of secretions [1,4,5]. According to published research, hearing loss, rhinosinusitis, and head‑and‑neck cancer are significant ENT problems [6,7]. According to the WHO, about 278 million individuals worldwide have moderate-to-severe bilateral hearing loss. The causes of hearing loss are preventable, and two-thirds of those affected live in developing nations [8,9]. Hearing loss was reported by 16.1% of individuals in the United States (US) between 2003 and 2004 [8,10]. In the adult US population, the prevalence of rhinosinusitis was reported to be 16% in 2004 [8,11]. More than 11 million people in the US have developed some form of permanent noise‑induced hearing loss [8,12-18]. Head‑and‑neck cancer is associated with morbidity, mortality, and high health costs in the US because it ranks as the 10th most common type of malignancy in the US and accounts for about 3% of all adult cancers [8,18-19]. ENT disorders frequently occur in the community [1,20]. There was limited available data regarding the knowledge and attitudes toward ENT-related problems locally. However, a previous study conducted among adults in Riyadh City showed that 2.3% of participants had excellent knowledge, while 18.4% had good knowledge. Moreover, the majority (79.4%) had poor knowledge [8]. ENT diseases may lead to various complications that affect daily living activities, including hearing loss, obstructive sleep apnea, and cardiopulmonary complications [1,20]. However, few studies have investigated students' knowledge and attitudes toward ENT-linked diseases in Saudi Arabia's schools and universities. Therefore, our study aimed to investigate and explore the knowledge of and attitudes toward common ENT problems among students in Makkah City, Saudi Arabia.

## Materials and methods

Study design, population, and sampling

This descriptive cross-sectional study was conducted at the College of Medicine at Umm Al-Qura University and at Makkah City high schools in Saudi Arabia between November 2021 and October 2022. The study population included male and female Makkah City high school students and Umm Al-Qura University students. Students from other regions and other universities were excluded. School names were gathered from the Ministry of Education's website for Makkah City and then divided into seven regions: North, South, East, West, Central, Bahrah, and Al-Jumum. The schools were organized alphabetically and selected randomly for each region using the Random.org website. University students were randomly selected according to gender and their college at Umm Al-Qura University. The sample was recruited from students who participated in the study. We estimated that the sample size required for the analysis would be 385, calculated by Epi Info version 2.1, considering the CI as 95% and the significance level (p-value) as 5% [21]. To account for potential data loss, the total sample size was increased to 400. However, data were eventually collected from 1,080 participants.

Data collection and instrument survey

Data were collected from completed self-administered Arabic-language surveys designed with a Google Forms questionnaire template and distributed online through social media. The study objectives were adopted from the reviewed literature and previously published questionnaires developed by board-certified otolaryngologists to cover the most common ENT-related diseases and misconceptions. It was then translated into Arabic-based language. It was also self-reported, preserved participants' privacy, and reviewed and modified by senior otolaryngologists to be understandable, simple, and reliable to the population [8]. The survey is divided into five sections. Each section contains a series of multiple-choice questions between two and five answer options. The first section includes the consent form. The second section contains questions identifying the exclusion criteria (e.g., do you live in Makkah City, do you study in educational institutions based in Makkah City). Participants who answered No were referred to the submit page. The third section elicits sociodemographic data, including gender, age, educational level, and nationality. The fourth section focuses on respondents' knowledge of ENT diseases. Respondents were first asked to provide information about their past medical and surgical history of otorhinolaryngology diseases, such as a past diagnosis of ENT problems and surgeries, as well as their use of ear cotton swabs and their use of antibiotics for upper respiratory tract infection symptoms, such as coughing and sneezing, their experience with influenza, whether they had been vaccinated for seasonal influenza as a protective measure and whether vaccination is recommended for people with diabetes. The fourth section also asks about respondents' knowledge about taking vitamin C to prevent and cure influenza, their understanding of vertigo (i.e., dizziness), whether hearing loss affects social life, the effects of continuous exposure to loud noises, whether inflammation of the middle or inner ear can lead to vertigo, whether ear pain is caused by middle ear inflammation, the proper way to handle nasal bleeding, the use of nasal congestion drops, whether surgery to remove adenoids leads to obesity or immune deficiency and whether continuous screaming can lead to vocal cord disturbances. Respondents answered all the questions with "yes," "no," or "I don't know." The last section included two questions: 1) "In case of sudden hearing loss or ear pain 1) I go to the hospital, 2) I use home remedies, 3) I ignore it, and 4) I don't know, and 2) "What is your source of information to answer the previous questions? 1) My doctor, 2) a pharmacist while dispensing my medication, 3) social media, 4) online searches, 5) community (i.e., family and friends), or 6) other sources.

Statistical analysis

The obtained data were analyzed statistically using SPSS version 23. The mean, SD, and significance utilizing the chi-square test were used to measure and compare. A significance level of <0.05 will be considered statistically significant in all analyses. The data were coded, entered into the SPSS program, and then analyzed using a modified Bloom's criterion scoring system. The final classification was made using the following categories: a score of 80%-100% was considered a good level of knowledge, a score of 50%-79% was considered a moderate level of knowledge, and a score less than 50% was considered a poor level of knowledge.

Ethical approval

The study was approved by the Institutional Research Board at Umm Al-Qura University, Makkah City, Saudi Arabia (HAPO-02-K-012-2021-09-732). All study participants were informed of the study's objectives and gave their consent, and were assured that their responses would be kept confidential.

## Results

An online survey targeted school students and medical students in Makkah City. A total of 1,080 individuals participated in this survey. Most respondents were older than 20 years (n=916, 84.8%). Female participants provided most of the completed questionnaires (n=766, 70.9%). Students with university degrees provided the majority of responses (n=849, 78.6%), while the majority of the questionnaires were completed by Saudi respondents (n=1022, 94.6%). Two hundred and ninety-five respondents (27.3%) reported a previous diagnosis of ear problems, 361 (33.4%) respondents reported a previous diagnosis of nose problems, and 262 (24.3%) respondents reported a previous diagnosis of throat problems. Moreover, 143 (13.2%) respondents reported having had previous ear, nose, and throat surgery (Table 1).

**Table 1:** Demographic data.

Variable	Category	Frequency (%)
Age	Less than 20	164 (15.2)
	More than 20	916 (84.8)
Gender	Male	314 (29.1)
	Female	766 (70.9)
Academic year	High school or less	231 (21.4)
	Bachelor’s degree or more	849 (78.6)
Nationality	Saudi	1022 (94.6)
	Non-Saudi	58 (5.4)
Previously diagnosed with ear problems	Yes	295 (27.3)
	No	785 (72.7)
Previously diagnosed with nose problems	Yes	361 (33.4)
	No	719 (66.6)
Previously diagnosed with throat problems	Yes	262 (24.3)
	No	818 (75.7)
Previous surgery concerning Ear, Nose, Throat.	Yes	143 (13.2)
	No	937 (86.8)

More than 50% of the respondents provided correct answers regarding the safety of using cotton swabs for ear cleaning (62.5%), the importance of getting the flu vaccine for prevention in normal individuals and individuals with comorbidities (67.4% and 51.8%, respectively), the importance of using vitamin C to prevent influenza (65.1%), the definition of vertigo (76.3%), the influence of hearing loss on social life (87%), the risk of hearing loss and vocal cord disorders with loud noises and screaming (60.1% and 71.9%, respectively), and knowledge of ear infection (83.1% and 60.4%, respectively). Further, more than 50% of participants provided correct answers to the questions about handling nose bleeds and using nasal drops (55.3% and 54.4%, respectively). However, fewer than 50% of the respondents provided correct answers to the questions about the efficacy of antibiotics in managing upper respiratory tract infection and the risk of developing obesity or immunodeficiency after tonsillectomy (34.1%, 41.4% and 23.9%, respectively) (Table 2).

**Table 2:** Questions about the attitude towards common otolaryngology issues.

Question	Yes	No	I don't know
Cotton swabs are an unsafe way to clean the ears	675 (62.5)	207 (19.2)	198 (18.3)
Antibiotics are not considered to treat upper respiratory infections (such as colds or colds)	368 (34.1)	471 (43.6)	241 (22.3)
It is recommended to get the flu vaccine annually as a preventive measure	728 (67.4)	176 (16.3)	176 (16.3)
It is recommended for patients with diabetes and high blood pressure to take the seasonal flu vaccine	559 (51.8)	77 (7.1)	444 (41.1)
Taking vitamin C treats and prevents influenza	703 (65.1)	150 (13.9)	227 (21.0)
Vertigo is another term for dizziness	824 (76.3)	179 (16.6)	77 (7.1)
Hearing loss can affect the social life of the owner	940 (87.0)	95 (8.8)	45 (4.2)
Constant exposure to loud and annoying noises or sounds may cause hearing loss	649 (60.1)	182 (16.9)	249 (23.1)
Some infections of the inner ear and middle ear cause dizziness	897 (83.1)	34 (3.1)	149 (13.8)
Not all earaches are necessarily considered middle ear infections	652 (60.4)	119 (11.0)	309 (28.6)
The correct way to deal with a nosebleed is to put the head back	308 (28.5)	597 (55.3)	175 (16.2)
The use of nasal congestion drops is considered safe for long-term use	156 (14.4)	588 (54.4)	336 (31.1)
Tonsillectomy may cause obesity	173 (16.0)	447 (41.4)	460 (42.6)
Tonsillectomy may cause immunodeficiency	373 (34.5)	258 (23.9)	449 (41.6)
Does constant screaming cause vocal cord disorders?	115 (10.6)	776 (71.9)	189 (17.5)

**Figure 1:**
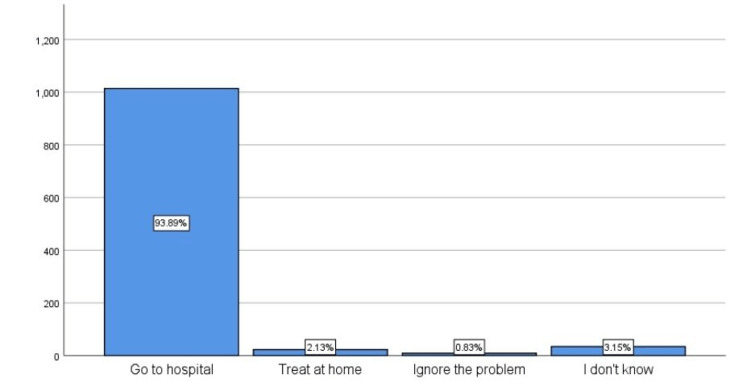
Participants' responses for appropriate action to take in case of sudden hearing loss or earacheIn case of sudden hearing loss and earache.

When participants were asked about the appropriate action to take in case of sudden hearing loss or earache, most of the participants reported that "going to the hospital" would be the appropriate action to take (93.89%) (Figure 1).

**Figure 2:**
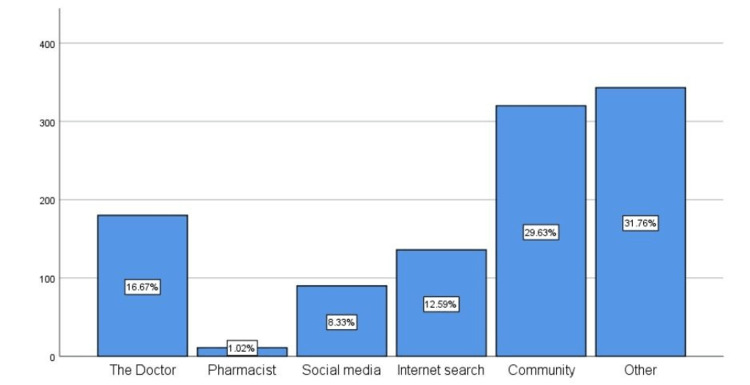
Sources of participants' answers to the previous questions.

Participants' sources of information and knowledge about ENT issues responding to the previously reported attitude in Table 2 included "other sources" (31.76%), followed by the community (family and friends) and doctors (29.63% and 16.67%, respectively). Fewer respondents reported their sources of information as a pharmacist, social media, and internet searches (1.02%, 6.33% and 12.59%, respectively) (Figure 2).

**Figure 3:**
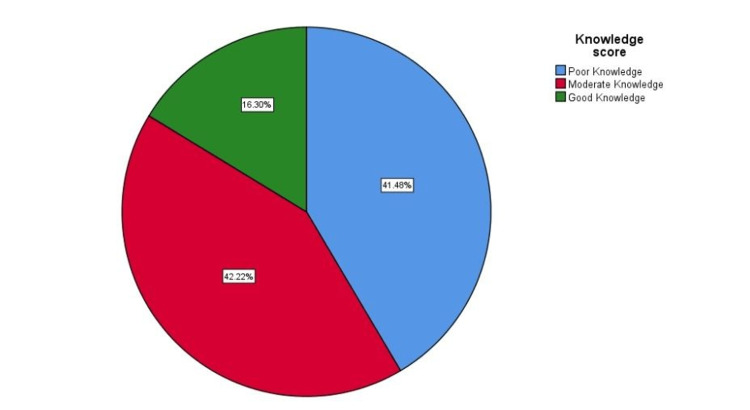
Knowledge score.

Most participants showed a moderate understanding of common ENT problems (42.22%) or a poor understanding (41.48%), while only 16.30% of the respondents showed a good level of knowledge concerning common ENT problems (Figure 3).

Table 3 shows the association between the level of knowledge and the participants' demographics. Overall, participants older than 20 years of age, females, and those with university level showed a good, moderate, and poor understanding of common ENT problems (p-values of <0.001, 0.004, and <0.001, respectively). Participants' educational levels and past ENT medical and surgical histories did not show a statistically significant association with their knowledge of ENT issues (p-values of 0.161, 0.192, 0.352, 0.183, and 0.133, respectively) (Table 3).

**Table 3:** Association between level of awareness and demographic data.

Variable	Level of knowledge			P-value
	Poor n (%)	Moderate n (%)	Good n (%)	
Age				
Less than 20	10 (6.1%)	62 (37.8%)	92 (56.1%)	0.000*
More than 20	166 (18.1%)	394 (43.0%)	356 (38.9%)	
Gender				
Male	51 (16.2%)	110 (35.0%)	153 (48.7%)	0.004*
Female	125 (16.3%)	346 (45.2%)	295 (38.5%)	
Academic year				
High school or less	16 (6.9%)	89 (38.5%)	126 (54.5%)	0.000*
Bachelor’s degree or more	160 (18.8%)	367 (43.2%)	322 (37.9%)	
Nationality				
Saudi	169 (16.5%)	436 (42.7%)	417 (40.8%)	0.161
Non-Saudi	7 (12.1%)	20 (34.5%)	31 (53.4%)	
Previously diagnosed with ear problems				
Yes	41 (13.9%)	124 (42.0%)	130 (44.1%)	0.352
No	135 (17.2%)	332 (42.3%)	318 (40.5%)	
Previously diagnosed with nose problems				
Yes	64 (17.7%)	161 (44.6%)	136 (37.7%)	0.192
No	112 (15.6%)	295 (41.0%)	312 (43.4%)	
Previously diagnosed with throat problems				
Yes	34 (13.0%)	120 (45.8%)	108 (41.2%)	0.183
No	142 (17.4%)	336 (41.1%)	340 (41.6%)	
Previous surgery concerning Ear, Nose, or Throat.				
Yes	30 (21.0%)	63 (44.1%)	50 (35.0%)	0.133
No	146 (15.6%)	393 (41.9%)	398 (42.5%)	

## Discussion

An online survey was completed by 1,080 high school and university students in Makkah City, Saudi Arabia. The results showed that 42.22% of participants were moderately aware of common ENT problems. The question that was most often answered correctly was about the appropriate action to take in case of sudden hearing loss and earache, which is to "go to the hospital" (93.89%). The question most often answered incorrectly was whether "tonsillectomy may cause immunodeficiency" (23.9%). This study showed that participants older than 20 had better knowledge of common ENT problems than those younger than 20. One of the potential explanations is that participants over 20 years of age may have a history of ENT-related issues that lead to increased awareness. Female respondents had a higher level of knowledge and awareness because, locally, they are caretakers of family health issues rather than males in general. We found few studies in the existing literature on the most common ENT problems among the population. However, our study showed that a greater percentage of participants had good knowledge of common ENT problems (16.30%) than a previous study conducted in Riyadh, Saudi Arabia, in 2020 (2.3%). In addition, most participants had good knowledge of the use of vitamin C in preventing influenza (65.1%), in contrast to the previous study, in which most participants showed poor knowledge (17.75%). Also, most participants showed good knowledge of vertigo, another term for dizziness (76.3%), compared to the previous study, in which only 19.9% showed good knowledge. In both studies, most participants showed good knowledge regarding the potential harms of using cotton earbuds [8]. In addition, a study conducted in Milan, Italy, in 2013 showed that most participants had good knowledge about the damage caused by cotton earbuds [11]. In the current and Italian studies, most participants showed good awareness regarding the action to be taken in case of sudden hearing loss. However, our study showed a better awareness level, as 93.89% of the participants reported that they would go to the hospital, while in the Italian study, only 80% gave this response [8,11].

Based on the current study's findings, a national awareness campaign is recommended to increase awareness of common ENT-related conditions. Further investigation is needed to establish the generalizability of the results of this study to other regions in Saudi Arabian. Moreover, this study shows that more completed questionnaires were received from high school students than university-level students, which is a possible limitation that also needs further investigation. As this is a survey-based study, recollection bias might be a limitation to consider. Furthermore, poor Internet access could have placed constraints on some people's ability to participate in the study, which might have skewed the results.

## Conclusions

According to the data obtained by this survey, 16.3% was the percentage of participants whose scores indicated that they had a good knowledge of ENT issues, which is higher than the results of prior studies. Participants older than 20, female participants, and participants with bachelor's degrees or university degrees had more substantial knowledge of common ENT problems than other participants. The highest number of correct answers (93.89%) was given to the question about what a person should do if they experienced hearing loss and earache (answer: "go to the hospital"). The highest number of incorrect answers was given to the question: "Does tonsillectomy lead to immunodeficiency?" (23.9%). Therefore, we recommend that the medical community make efforts to increase the knowledge of the general population about common ENT problems.
